# CD8^+^ T cell heterogeneity and function in lupus nephritis: a focused review

**DOI:** 10.3389/fimmu.2026.1844836

**Published:** 2026-08-13

**Authors:** Zhaomin Mao, Luoyi Wang, Jing Chen, Ming Guan

**Affiliations:** 1 Department of Laboratory Medicine, Huashan Hospital, Fudan University, Shanghai, China; 2 Division of Nephrology, Huashan Hospital, Fudan University, Shanghai, China; 3 National Clinical Research Center for Aging and Medicine, Huashan Hospital, Fudan University, Shanghai, China

**Keywords:** biomarker, CD8+ T, lupus nephritis, pathogenesis, subset

## Abstract

Lupus nephritis (LN) is a severe manifestation of systemic lupus erythematosus marked by immune complex deposition and tissue-specific inflammation. Advances in single-cell transcriptomics and high-dimensional immune profiling have revealed that CD8^+^ T cells form a highly heterogeneous and compartment-dependent landscape across peripheral blood, kidney, and urine. This review aims to delineate how compartment-specific phenotypes and signaling programs of CD8^+^ T cells collectively shape the immunopathogenesis of lupus nephritis (LN). We synthesize current evidence into a unified blood-kidney-urine circuit model, in which systemic priming of circulating CD8^+^ T pools is followed by renal recruitment, tissue retention and functional reprogramming of kidney-infiltrating populations, cytotoxic and inflammatory tissue injury, and subsequent release of intrarenal immune signatures into urine. Circulating CD8^+^ T cells include IFN-I–primed naïve-like, effector-memory, and cytotoxic subsets that contribute to systemic immune activation. Within the kidney, infiltrating CD8^+^ T cells display pronounced clonal expansion, tissue-resident and exhaustion-associated phenotypes, and metabolic reprogramming, all of which correlate with local inflammation and histopathologic severity. Urine-derived CD8^+^ T cells, increasingly recognized as a non-invasive proxy for intrarenal immunity, recapitulate key transcriptional programs of kidney-infiltrating populations and provide a dynamic readout of ongoing renal injury. Across these anatomical niches, CD8^+^ T-cell effector and regulatory functions are shaped by pivotal pathways, including type I IFN conditioning, JAK–STAT signaling, NLRP3 inflammasome activation, mitochondrial stress responses, and modulation by immune checkpoints. Collectively, these insights highlight CD8^+^ T cells as an important effector and regulatory component within the broader multicellular immune network of LN and suggest that resolving their compartmental heterogeneity and signaling circuits may accelerate the development of mechanistically grounded biomarkers and targeted immunomodulatory strategies.

## 1 Introduction

Systemic lupus erythematosus (SLE) is a chronic autoimmune disease characterized by widespread inflammation and immune-mediated damage (1–4). Lupus nephritis (LN), one of its most severe complications, develops in about half of SLE patients within five years of diagnosis and often leads to end-stage renal disease (ESRD), despite immunosuppressive therapy (5–13). Thus, understanding the pathogenesis of LN is crucial for developing targeted therapies.

Lupus nephritis (LN), a prototypical autoimmune kidney disease, is characterized by immune complex deposition and immune cell infiltration. Among the infiltrating lymphocytes, CD8^+^ T cells have emerged as pivotal contributors to both tissue injury and immune regulation (14–17). Although historically overshadowed by CD4^+^ T cells and B cells, advances in single-cell RNA sequencing (scRNA-seq), flow cytometry, mass cytometry, T cell receptor sequencing, and immunostaining have highlighted distinct CD8^+^ subsets in peripheral blood, kidney tissue, and urine. These subsets include cytotoxic effector T cells, tissue-resident memory T cells (TRM), exhausted-like PD-1^+^ populations, and others (18–23). Importantly, lupus nephritis is driven by a highly interconnected multicellular immune network involving immune complexes, B cells, plasma cells, CD4^+^ T helper subsets, T follicular helper cells, regulatory T cells, myeloid cells, neutrophils, endothelial cells, podocytes, and tubular epithelial cells (24, 25). Within this complex pathogenic ecosystem, CD8^+^ T cells should be viewed as an effector and regulatory node that integrates systemic immune activation with local renal injury rather than acting as an isolated or dominant driver of disease (24–26). Understanding the various CD8^+^ T-cell subsets involved in LN and their molecular drivers is crucial for the development of targeted therapies.

This review provides a comprehensive overview of CD8^+^ T-cell diversity in lupus nephritis and discusses its roles in disease pathogenesis, as well as its potential implications for diagnosis and therapeutic intervention. We further propose a compartmentalized CD8^+^ T-cell model of lupus nephritis, linking circulating effector reservoirs, kidney-resident pathogenic tissue-resident memory (TRM) populations, and urinary immune readouts.

## 2 A core compartmental mechanistic framework of CD8^+^ T cells in lupus nephritis

**Figure 1:**
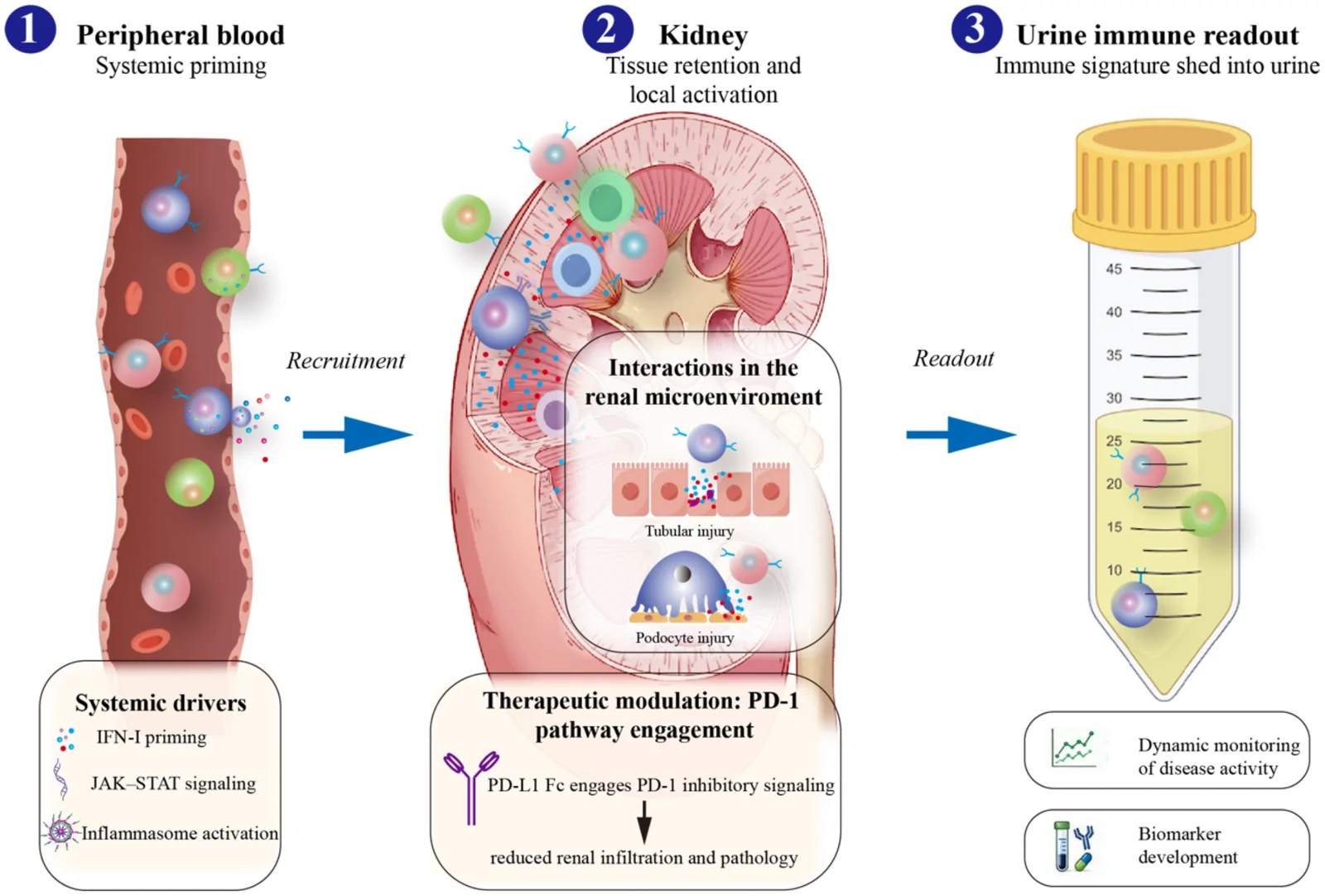
Proposed blood–kidney–urine CD8^+^ T-cell circuit in lupus nephritis. Systemic inflammatory cues promote circulating CD8^+^ T-cell activation, effector-memory remodeling, and recruitment to the kidney. Within the renal microenvironment, CD8^+^ T cells undergo tissue retention, local activation, and functional reprogramming, contributing to tubular and podocyte injury through cytotoxic and inflammatory pathways. Urinary CD8^+^ T cells and CD8-associated immune signatures provide a non-invasive readout for disease monitoring and biomarker development. Therapeutic engagement of PD-1-mediated inhibitory signaling by PD-L1 Fc may restrain hyperactive PD-1^+^CD8^+^ T-cell responses and reduce renal inflammation (by Figdraw and Adobe Illustrator).

Rather than representing isolated immune populations in separate anatomical sites, CD8^+^ T cells in lupus nephritis can be conceptualized as a dynamic blood-kidney-urine circuit (24). In this framework, systemic inflammatory cues, particularly type I interferon and JAK-STAT signaling, prime circulating CD8^+^ T cells toward effector-memory, cytotoxic, and partially exhausted phenotypes (24, 27) (**Figure 1**). These circulating CD8^+^ T-cell pools may then serve as a reservoir for renal recruitment through chemokine axes such as CXCR3-CXCL9/10/11, CCR5-related pathways, and CX3CR1-CX3CL1 signaling (24, 28). Once within the kidney, local antigen presentation, inflammatory cytokines, metabolic stress, and tissue-derived danger signals promote clonal expansion, tissue retention, and differentiation into tissue-resident memory or effector-exhausted states (21). These renal CD8^+^T-cell populations contribute to tissue injury through granzyme/perforin-mediated cytotoxicity, IFN-γ production, inflammasome activation, and amplification of tubular and podocyte damage (21, 29). Finally, urinary CD8^+^ T cells and CD8-associated transcripts provide a non-invasive readout of this intrarenal immune circuit (30). Therefore, the central theme of this review is that CD8^+^ T cells in LN should be understood not simply as compartment-specific subsets, but as interconnected immune states that link systemic immune activation, renal tissue injury, and urinary biomarker discovery.

## 3 The timeline of CD8+ T-cell subtypes discovery in lupus nephritis

CD8^+^ T cells, pivotal mediators that exert cytotoxic effects, play essential roles in immune surveillance and combating infections and autoimmunity (31, 32). Early studies in LN focused on immune-complex injury, with T cells identified as important infiltrators. Although the precise pathogenesis remains unclear, growing evidence suggests that CD8^+^ T cells may be an important determinant of the pathogenesis and progression of LN. In early studies during the 1980s–1990s, CD8^+^ T cells were reported to account for large proportion of the mononuclear cells infiltrating the kidneys of LN patients (14). CD8^+^ T cells are more prevalent in LN compared to other primary glomerulonephritis, underscoring their importance in LN pathogenesis (15, 16). Meanwhile, the presence of CD8^+^ T cells was detected in the infiltrating inflammatory cells of the kidney in the lupus-prone mice model in 1996 (33). Over the last 25 years, as phenotyping improved, the researchers utilized flow cytometry, immunostaining, T cell receptor sequencing, or mass cytometry to reveal the presence of different presentation and function of CD8+ T cell and their subtypes in the peripheral blood, urine, and kidney biopsy tissues of lupus nephritis patients and mouse models, such as CD8^+^ effector, effector-memory (TEM) cells, tissue resident memory T cells (TRM), etc. (21, 23, 27, 34–39). Translational clinical observations noted that cytotoxic degranulation markers (e.g., CD107a/LAMP-1) on CD8^+^ T cells are decreased in SLE/LN blood and increased in kidneys. These compartment-specific findings support the concept that CD8^+^ T-cell cytotoxic activity may contribute to renal tissue injury in LN. Conversely, the discovery of regulatory CD8^+^ T cells (and CD103^+^ subsets) and their broad anti-inflammatory activities suggests that not all CD8^+^ T cells are pathogenic. The function of CD8^+^ T cells depends on their subtype, activation signals, and tissue context (37, 40, 41).

A pivotal leap came with single-cell RNA-seq (scRNA-seq) atlases of LN kidneys, which mapped immune ecosystems at unprecedented resolution. The landmark Accelerating Medicines Partnership (AMP) study by Arazi et al. in 2019 catalogued renal leukocyte subsets—including multiple CD8^+^ states—with robust interferon-response signatures and chemokine axes linking kidney and urine, establishing urine as a feasible surrogate for kidney immune readouts (24). Subsequent single-cell and TCR-seq datasets further emphasized clonally expanded, cytotoxic‐skewed CD8^+^ T cells and tissue-resident memory (TRM) phenotypes in kidneys and urine (18, 21, 35, 42, 43).

**Figure 2:**
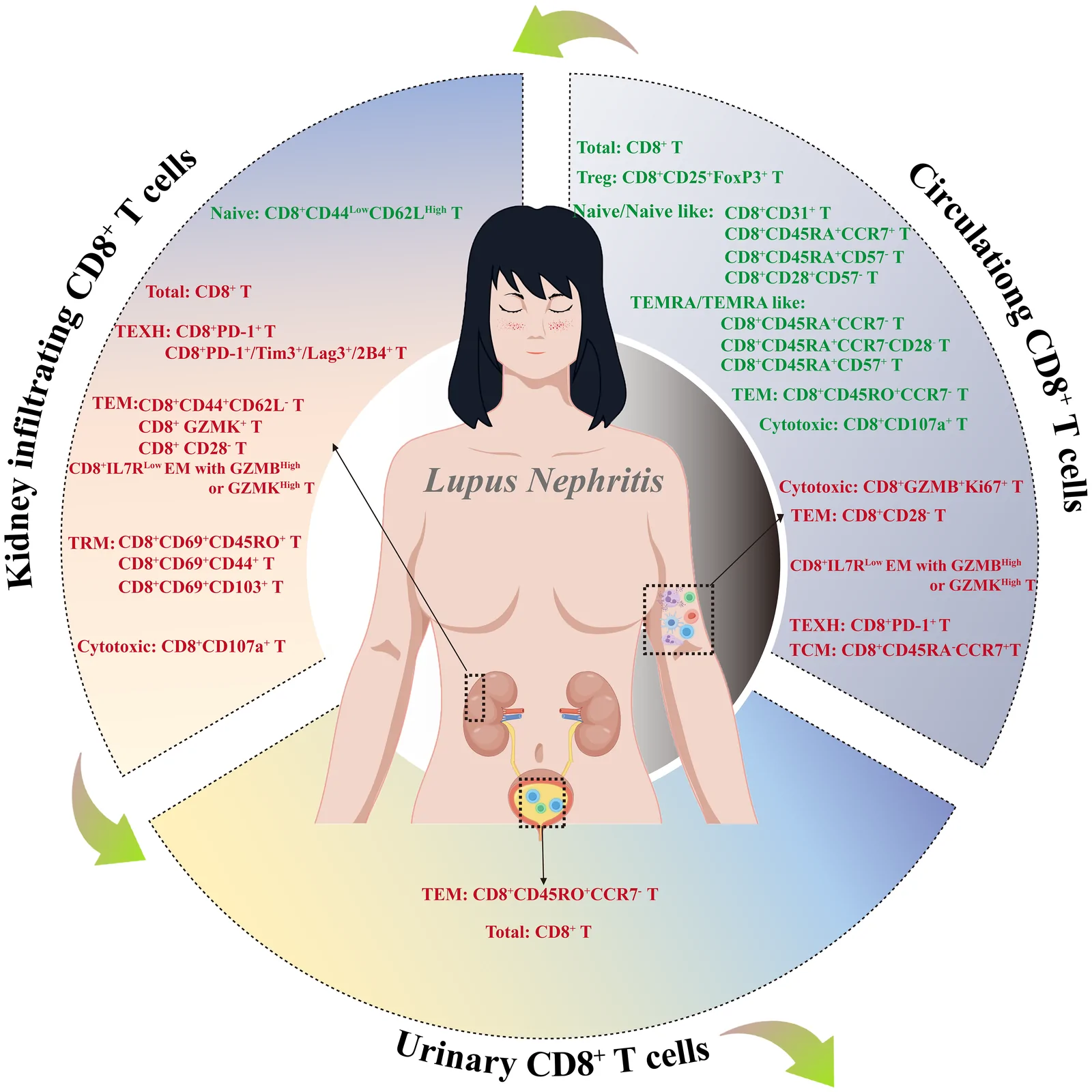
Expression pattern of various CD8+ T-cell subtypes in Lupus Nephritis. Red represents an increase, while green represents a decrease. TRM, tissue-resident memory T cells; TEM, effector memory T cells; TCM, central memory T cells; Treg, regulatory T cells; TEXH, exhausted T cells; TEMRA, effector memory re-expressing CD45RA^+^ T cells (by Figdraw and Adobe Illustrator).

In summary, breakthrough studies over the past decade have repositioned T cells from mere bystanders to important players in the pathogenic process of lupus nephritis. Distinct CD8^+^ T cell subsets from different tissues exert diverse yet essential roles in the progression. The expression change of CD8^+^ T cells and their subtypes in lupus nephritis is depicted in detail in **Figure 2** and **Table 1**.

**Table 1:** CD8+ T cell subsets in lupus nephritis.

CD8^+^ T-cell subset	Species (Human/Mouse)	Key findings	Citation
Total			
CD8^+^	Human	Predominant kidney-infiltrating subset; Correlated with disease activity and therapeutic response; increase in the urine of active stage; the absolute number decrease in peripheral blood	(15, 17, 22, 23, 35, 53, 58)
Naive/Naive like			
CD8^+^CD44^Low^CD62L^High^	Mouse	Decrease in kidney	(39)
CD8^+^CD31^+^	Human	Decrease in peripheral blood	(22)
CD8^+^CD45RA^+^CCR7^+^	Human	Decrease in peripheral blood	(22)
CD8^+^CD45RA^+^CD57^−^	Human	Decrease in peripheral blood	(22)
CD8^+^CD28^+^CD57^−^	Human	Decrease in peripheral blood	(22)
Cytotoxic			
CD8^+^CD107a^+^	Human	Decrease in peripheral blood and increase in kidney; associated with disease activity	(37)
CD8^^+^^GZMK^+^IFN-γ^+^	Human	Increase in kidney and promote the differentiation of atypical B cells	(20)
CD8^+^GZMB^+^Ki67^+^	Human	Associated with cytotoxic and IFN-I-related immune activation	(54)
TRM			
CD8^+^CD69^+^CD103^+^	Mouse/Human	Contribute to renal inflammation and damage	(27, 52)
CD8^+^CD69^+^CD44^+^	Mouse	Increase in kidney, promote podocyte injury	(21)
CD8^+^CD69^+^CD45RO^+^	Human	Increase in kidney, promote podocyte injury	(21)
TEM			
CD8^+^CD28^-^	Human	Increase in peripheral blood and kidney, promote inflammation	(35, 36)
CD8^+^CD45RO^+^CCR7^-^	Human	Decrease in peripheral blood/active renal involvement and increase in urine	(34)
CD8^+^GZMK^+^	Human	The dominant T cell subset that mediates renal injury	(20)
IL7R^low^ EM CD8^+^ T cells with GZMK^High^ or GZMB^High^	Human	Accumulate in the peripheral blood and kidney of LN; associate with disease activity	(44)
TCM			
CD8^+^CD45RA^-^CCR7^+^	Human	Increase in peripheral blood	(22)
Treg			
CD8^+^CD25^+^FoxP3^+^	Human	Decrease in peripheral blood	(40)
CD8^+^CD103^+^	Mice	Relieve disease severity and improve prognosis	(45, 46)
TEXH			
CD8^+^PD1^+^/Tim3^+^/Lag3^+^/2B4^+^	Mouse/Human	Increase in kidney	(39)
CD8^+^PD1^+^	Mice/Human	Increase in peripheral blood and kidney	(22, 39)
TEMRA/TEMRA like			
CD8^+^CD45RA^+^CCR7^−^	Human	Decrease in peripheral blood	(22)
CD8^+^CD45RA^+^CCR7^−^CD28^−^	Human	Decrease in peripheral blood	(22)
CD8^+^CD45RA^+^CD57^+^	Human	Decrease in peripheral blood	(22)

TRM, tissue-resident memory T cells; TEM, effector memory T cells; TCM, central memory T cells; Treg, regulatory T cells; TEXH, exhausted T cells; TEMRA, effector memory re-expressing CD45RA^+^ T cells.

## 4 Peripheral blood CD8^+^ T cell subsets: roles and functions in circulation

Peripheral blood CD8^+^ T-cell subsets in lupus nephritis (LN) display a complex and dynamic landscape that shapes both immune activation and regulatory process. These subsets encompass cytotoxic effector and effector-memory pools, exhausted PD-1^+^ cells, CD8^+^ Tregs and others, each of which may play distinct roles in disease progression. The following sections will delve into the characteristics and functions of these subsets, exploring their impact on the immunopathogenesis of LN and their potential as therapeutic targets.

### 4.1 Effector cytotoxic and effector-memory pools (Tc1/TEM/TEMRA)

In peripheral blood, effector-memory and TEMRA-related CD8^+^ T-cell phenotypes in LN appear to be remodeled rather than uniformly expanded. Flow-cytometric studies have reported reductions in several CD45RA^+^/CCR7-defined or senescence-associated terminally differentiated CD8^+^ subsets, whereas selected cytotoxic or inflammatory CD8^+^ programs, including GZMB, PRF1, GNLY, and GZMK-associated signatures, may be increased in specific disease contexts. These findings suggest that peripheral cytotoxic and effector-memory pools are dynamically redistributed according to disease activity, marker definition, and tissue recruitment (22, 37, 38). Recent TCR repertoire analyses further support this dynamic model by showing that CD8^+^ T-cell clonal expansion and immune dysregulation accompany the progression from systemic lupus erythematosus to lupus nephritis, suggesting that CD8^+^ T-cell remodeling may evolve alongside renal involvement rather than representing a static immune abnormality (26).

Single-cell work combined microarray analyses and other technologies complement older flow-cytometric observations by providing transcriptional granularity and cross-tissue linkage. These data have refined this picture: IL7R^low^ effector-memory CD8^+^ subsets in blood and kidney with heightened cytotoxic/inflammatory programs (GZMB, GZMK, PRF1, GNLY) serve as circulating reservoirs for renal recruitment (20, 44).

### 4.2 Exhausted/PD-1^+^ CD8^+^ T cells

The PD-1 marker is not a universal indicator of dysfunction on CD8^+^ T cells in LN; rather, it marks an adaptive response to chronic autoantigen exposure. PD-1^+^CD8^+^ T cells are significantly increased in circulation during active LN (22). These cells exhibit a hybrid phenotype that combines markers of exhaustion with strong effector signatures. Single-cell RNA-seq in mice reveals that PD-1^+^CD8^+^ T cells segregate into exhausted-effector states, many of which show clonal expansion, indicating antigen-driven activation rather than passive exhaustion (19). Thus, circulating PD-1^+^ CD8^+^ T cells may serve as a peripheral indicator of chronic antigenic stimulation and systemic immune activation, while their pathogenic potential and regulation through the PD-1/PD-L1 axis are more directly demonstrated in renal tissue and lupus-prone mouse models.

### 4.3 CD8^+^ Treg cells

In patients with lupus nephritis (LN) and lupus-prone mouse models, peripheral CD8^+^ regulatory T cells (CD8^+^ Tregs) form a crucial counter-regulatory arm that restrains humoral and cellular autoimmunity, but they are numerically and/or functionally insufficient in active disease. Clinical data from childhood class III/IV LN show that intravenous methyl-prednisolone pulses are followed by an expansion of circulating CD8^+^ Tregs whose frequencies negatively correlate with disease activity and proteinuria, suggesting that restoration of this compartment contributes to clinical improvement (40).

Mechanistic work using lupus-prone mice demonstrates that peripheral CD8^+^ T cells can be converted ex vivo into TGF-β–induced CD8^+^CD103^+^ induced Tregs (CD8^+^CD103^+^ iTregs), which suppress B-cell activation and autoantibody production after adoptive transfer, attenuate glomerular endothelial cell injury by blocking p38 MAPK and NF-κB signaling and ameliorate chronic GVHD–associated lupus nephritis. These effects are critically dependent on high expression of the ectonucleotidase CD39 on CD8^+^ iTregs and adenosine generation (45–47). Thus, strategies that increase CD8^+^ Treg number or function in the peripheral blood represent a promising therapeutic avenue alongside broader Treg-based cell therapies that have already shown reductions in anti-dsDNA titers and albuminuria in SLE (48, 61).

In summary, peripheral blood in active LN shows context-dependent remodeling of cytotoxic, effector-memory, exhausted-like, and regulatory CD8^+^ T-cell states. PD-1 expression may identify chronically stimulated CD8^+^ T cells, whereas PD-1-mediated inhibitory signaling may serve as a negative regulatory mechanism. These patterns suggest that blood CD8^+^ signatures may provide complementary biomarkers and potential therapeutic clues in LN.

## 5 Renal CD8^+^ T cell subsets: roles and functions within the kidney

The kidney is a critical effector site for CD8^+^ T-cell activity in lupus nephritis, where multiple CD8^+^ T-cell states contribute to renal inflammation, tissue injury, and disease progression. These populations include tissue-resident memory T cells (TRM), cytotoxic effector T cells, PD-1^+^ exhausted-like or effector-exhausted cells, and clonally expanded kidney-infiltrating CD8^+^ T cells. Single-cell RNA sequencing and T-cell receptor sequencing studies have revealed substantial heterogeneity and clonal expansion of kidney-infiltrating CD8^+^ T cells, suggesting dynamic transitions among effector, tissue-retained, and exhaustion-associated states within the renal inflammatory microenvironment (18, 39, 49).

Spatially, CD8^+^ T cells in LN are not uniformly distributed throughout the kidney. Earlier immunohistochemical studies showed that CD8^+^ T lymphocytes predominate among periglomerular infiltrating cells in class III/IV LN and are linked to poor response after induction therapy (17). Additional studies demonstrated that expanded CD8^+^ T-cell clonotypes traffic into the lupus kidney, supporting antigen-driven or locally selected T-cell accumulation (35). More recent quantitative analyses of LN biopsies further confirmed that CD8^+^ lymphocytes were associated with both acute renal failure and progression to ESRD and can be detected in intraglomerular, periglomerular, and interstitial regions, with higher CD8^+^ counts than CD4^+^ cells across these areas and correlations with lesion severity (50, 51).

At the subset level, available evidence supports a model of subset-biased spatial enrichment rather than strict anatomical compartmentalization. CD8^+^ TRM cells, typically characterized by CD69 and CD103 expression, are expanded in LN kidneys (27). Cytotoxic CD8^+^ T cells, including CD107a^+^/LAMP-1^+^ populations and granzyme/perforin-expressing subsets, are particularly relevant to tubulointerstitial or peritubular inflammatory lesions, where they may interact with injured tubular epithelial compartments and contribute to local tissue damage (37). PD-1^+^ CD8^+^ T cells show context-dependent behavior: in some murine studies they resemble metabolically and functionally exhausted kidney-infiltrating T cells, whereas recent data indicate that PD-1^+^ CD8^+^ cells may also retain hyperactivated cytotoxic and inflammatory programs and localize near glomeruli, renal tubules, and Bowman’s capsule (19, 39). Therefore, CD8^+^ T-cell localization in LN should be understood as a spatially biased and disease-stage-dependent process, shaped by local antigen exposure, chemokine-guided recruitment, tissue retention, and inflammatory remodeling. The following sections summarize the major renal CD8^+^ T-cell subsets and their functional relevance to kidney pathology.

### 5.1 Tissue-resident memory CD8^+^ T cells

The most consistent renal CD8^+^ T cell signature in recent years is the accumulation of CD69^+^CD103^+^ TRM cells in LN kidneys. These cells are poised for fast effector responses, resist egress, and can persist long-term within the inflamed niche. They displayed enhanced cytotoxicity, maintained chronic kidney inflammation, and promoted podocyte/tubule injury (27, 52). ScRNA-seq and TCR-seq show that these TRM cells have clonally expanded cytotoxic gene programs (GZMB/PRF1^+^), interferon-response priming, and chemokine receptor patterns indicative of renal retention. Functional mouse studies show that interfering with JAK/STAT signaling diminishes TRM survival and improves renal function. Similarly, targeting TRM in models of glomerular disease, including lupus-like nephritis, has been shown to protect podocytes and reduce glomerular injury (27).

Emerging data implicate inflammasome activity (e.g., NLRP3, caspase-1, GSDMD) within renal CD8^+^ TRM cells as a driver of sustained effector function and local tissue damage. These findings position NLRP3 as a potential therapeutic target for controlling TRM pathogenicity in LN (52).

### 5.2 Cytotoxic effector CD8^+^ T cells

Immunohistochemistry and histopathology associate dense tubulointerstitial CD8^+^ infiltrates with renal impairment and poor outcomes in LN. Cytotoxic degranulation (CD107a^+^) is observed within kidney tissue. Together these data argue that CD8-mediated cytotoxicity contributes directly to tubular and podocyte injury, and that its tissue localization (especially interstitium) carries prognostic weight (37, 53). Moreover, the infiltration of IL7R^low^ EM CD8^+^ T cells with GZMK^High^ or GZMB^High^ effector memory cells in the kidney was associated with the treatment outcomes of lupus nephritis (44). CD8^+^GZMB^+^Ki67^+^ cytotoxic T cells were associated with necrotizing lesions and impaired renal recovery (54).

### 5.3 Exhausted/PD1^+^ CD8^+^ T cells

PD-1^+^ CD8^+^ T cells represent a context-dependent renal CD8^+^ T-cell population in lupus nephritis. Although PD-1 is commonly regarded as an exhaustion-associated marker in cancer and chronic infection, its expression in LN does not necessarily indicate terminal dysfunction. Recent evidence shows that PD-1^+^ CD8^+^ T cells are enriched in LN patients and lupus-prone NZB/W F1 mice. In the kidneys of severely diseased NZB/W F1 mice, single-cell RNA sequencing further identified heterogeneous PD-1^+^ CD8^+^ T-cell subsets with diverse effector programs and robust TCR clonal expansion, supporting an antigen-driven, hyperactive effector-like state rather than passive exhaustion (18, 19, 39). Therapeutically, PD-L1 Fc fusion protein suppressed hyperactive PD-1^+^ CD8^+^ T-cell responses, reduced IFN-γ production, and improved proteinuria and renal pathology in lupus-prone mice (19).

### 5.4 Interactions with renal stroma/myeloid/inflammatory cells

Single-cell analyses of LN kidneys highlight interactions between CD8^+^ T cells, dendritic cells, macrophages, and injured tubular epithelium. These interactions, driven by IFN-responsive programs, antigen presentation, and chemokine signaling (e.g., CXCR3/CXCL9/10/11, CX3CR1/CX3CL1), likely guide T-cell recruitment and retention in the kidney. Damage-associated signals from tubules and podocytes further propagate cytotoxic loops. These datasets also provide a mechanistic explanation for the observed concordance between urinary and renal immune transcripts—useful for non-invasive monitoring (24). Besides, studies have shown that GZMK+ CD8+ T cells in the kidneys of lupus nephritis patients can promote the differentiation of atypical B cells. This effect is related to the excessive activation of CD8^+^ GZMK^+^ T cells, which may provide new ideas for the treatment of lupus nephritis (20). The *in vitro* assay indicated that the TEM cells (IL7R^low^ EM CD8^+^ T cells with GZMK^High^ or GZMB^High^) could induce neutrophil extracellular trap (NET), which is a crucial inflammatory pathway during the onset of lupus nephritis (44).

In summary, renal CD8^+^ TRM and cytotoxic effectors are important effectors of tissue damage. Their survival and function are shaped by JAK/STAT and inflammasome pathways and tempered by PD-1 signaling, of which density and phenotype correlate with clinical severity and prognosis.

## 6 Urinary CD8^+^ T cell subsets: roles, functions, and biomarker potential

Urine serves as a non-invasive window into kidney-resident immunity, with early studies showing that urinary T cells, particularly CD8^+^ T cells (refer to intact CD8^+^ T lymphocytes detected in urine sediment by flow cytometry or single-cell-based approaches), reflect renal disease activity (55, 56). A low urinary CD4:CD8 ratio is characteristic for LN and helps distinguish it from other nephritides. Effector-memory CD8^+^ T cells are detectable in urine during flares, mirroring blood-to-kidney migration patterns and supporting the concept that urine captures ongoing renal immunology (30). Importantly, urinary CD8^+^ T cells should be interpreted as a cellular biomarker that complements, rather than replaces, conventional clinical and serological markers such as proteinuria, serum creatinine/eGFR, anti-dsDNA antibodies, and complement levels. Conventional markers are clinically indispensable but may not always distinguish ongoing intrarenal inflammation from residual proteinuria or chronic structural damage. In this context, urinary CD8^+^ T-cell enumeration may provide an inflammation-oriented, non-invasive readout of active renal immune-cell trafficking, particularly when interpreted together with urinary chemokines, proteinuria trends, renal function, and serological activity markers (23).

The AMP scRNA-seq atlases strengthened this paradigm by showing high correlation between immune gene expression in urine and kidney tissue, positioning urinary immune cells as surrogates for renal immune states. Single-cell analyses revealed overlapping immune activation states between kidney and urine, including cytotoxic and tissue-resident memory–like transcriptional programs, supporting urine as a partial non-invasive surrogate of intrarenal CD8+ T-cell activity (24).

Mechanistically, urinary CD8^+^ cells likely reflect both egress from inflamed parenchyma and transient luminal trafficking during active injury. Clinically, integrating urinary CD8+ readouts with chemokine-axis markers (e.g.,CCR5 and CXCR3) and damage markers may improve flare detection and response assessment, complementing proteinuria, which often lags behind immunologic activity (23, 57, 58). To some extent, urinary proteomics can predict the infiltration of cells within the kidneys. Integrated urine proteomics and renal single-cell genomics suggest that IFN-γ–associated urinary chemokine signatures may partly reflect intrarenal CD8^+^ T-cell infiltration and activation.

In summary, urinary CD8^+^ T cells, enriched for effector-memory/cytotoxic programs, mirror kidney immune states, correlate with disease activity, and offer a practical biospecimen for longitudinal monitoring in LN (24, 30).

## 7 Cross-compartmental synthesis and clinical implications

The value of a compartmental framework lies in its ability to distinguish shared CD8^+^ T-cell molecular programs from their distinct biological interpretations across tissue niches. Across peripheral blood, kidney, and urine, conserved modules--including type I interferon responsiveness and JAK-STAT signaling, cytotoxic effector gene expression, chemokine receptor programs, and immune checkpoint pathways—are repeatedly observed. However, the functional meaning of these shared signatures is context-dependent. In peripheral blood, they primarily reflect systemic immune activation and define a circulating reservoir of CD8^+^ T cells available for tissue recruitment. In the kidney, these programs correspond to locally retained and reprogrammed effector populations embedded within a pro-inflammatory microenvironment, where they directly interact with glomerular, tubular, stromal, and immune cells to mediate tissue injury. In urine, these signatures represent shed or trafficking-derived immune components that serve as a partial, non-invasive surrogate of intrarenal immune activity.

## 8 Conclusion

CD8^+^ T cells in lupus nephritis form a dynamic, compartment-linked immune circuit rather than isolated tissue-resident populations. Across blood, kidney, and urine, shared molecular programs are shaped by distinct microenvironmental contexts, driving transitions from systemic activation to renal effector differentiation and urinary immune readouts. This framework reconciles previously fragmented observations and highlights that disease activity reflects coordinated CD8^+^ T-cell state transitions across compartments. It further supports integrated biomarker strategies and therapeutic approaches targeting both CD8^+^ T-cell effector functions and their trafficking and reprogramming pathways.

## 9 Future perspectives and therapeutic targeting

1. Checkpoint Tuning for Hyperactive CD8^+^ T Cells. Preclinical data indicate that activating PD-1 signaling, rather than blocking it, can restrain hyperactive CD8^+^ T cell responses and attenuate nephritis. This opens the possibility of exploring PD-1 agonists or bispecific antibodies with renal pharmacodynamic monitoring, such as urinary CD8^+^ metrics (19).
2. Cytotoxicity dampening. Future strategies may explore ways to dampen excessive cytotoxic CD8^+^ T-cell activation by targeting upstream inflammatory cues such as type I interferon, JAK–STAT signaling, or granzyme-associated pathways, while preserving antiviral immune surveillance (20, 59, 60).
3. Harnessing regulatory CD8^+^ subsets. CD8^+^ regulatory T cells remain underexplored in LN. Expanding or adoptively transferring such cells may complement TRM/cytotoxic control—an area ripe for mechanistic and translational work (46, 61).
4. Biomarker-integrated trials. Incorporating urinary CD8^+^ readouts and spatial kidney immunophenotyping as trial endpoints will help resolve pharmacodynamics and identify responders for CD8-targeted therapies.
